# The Cutaneous Immune Microenvironment in Selected Inflammatory Skin Diseases: Linking Histopathology, Mechanisms, and Targeted Therapy

**DOI:** 10.3390/dermatopathology13020022

**Published:** 2026-05-10

**Authors:** Andreea Cătălina Tinca, Andreea Raluca Cozac-Szoke, Ovidiu Simion Cotoi

**Affiliations:** 1 Pathophysiology Department, George Emil Palade University of Medicine, Pharmacy, Science and Technology, 540142 Targu Mures, Romania; andreea-catalina.tinca@umfst.ro (A.C.T.); ovidiu.cotoi@umfst.ro (O.S.C.); 2 Pathology Department, Clinical County Hospital Mureș, 540136 Targu Mures, Romania

**Keywords:** atopic dermatitis, psoriasis, hidradenitis suppurativa, vitiligo, immune microenvironment, dermatopathology

## Abstract

Inflammatory skin diseases are characterized by complex interactions between immune pathways, epidermal barrier function, and environmental triggers, leading to distinct clinical and histopathological features. This narrative review aims to integrate current knowledge on the cutaneous immune microenvironment across major inflammatory skin diseases, including atopic dermatitis, psoriasis, hidradenitis suppurativa, and vitiligo. A comprehensive literature search was conducted using PubMed, Web of Science, and Scopus, focusing on studies published between 2021 and early 2026. The findings highlight disease-specific immune signatures, such as Th2-driven inflammation in atopic dermatitis, IL-23/Th17 axis activation in psoriasis, neutrophil-dominated responses in hidradenitis suppurativa, and cytotoxic T-cell-mediated melanocyte destruction in vitiligo. These molecular pathways are closely reflected in histopathological patterns, emphasizing the link between morphology and immunopathogenesis. Advances in targeted therapies, including biologics and Janus kinase inhibitors, demonstrate the clinical relevance of these pathways and support a transition toward mechanism-based treatment strategies. Dermatopathology is increasingly contributing to precision medicine approaches by supporting correlations between tissue features, immune pathways, and potential therapeutic targets. This review provides a framework for improved disease stratification and for the development of personalized treatment strategies in inflammatory skin diseases.

## Simple Summary

Inflammatory skin diseases such as atopic dermatitis, psoriasis, hidradenitis suppurativa, and vitiligo are common conditions that impact patients’ quality of life. These diseases are driven by complex interactions between immune cells and the skin. Recent research has shown that the skin is not merely a mechanical barrier but an active immune organ, where various cells and molecules interact to initiate and sustain inflammation. This review explains how these immune processes are reflected in histopathological changes and how they relate to modern therapeutic approaches.

## 1. Introduction

Inflammatory skin diseases represent a broad group of chronic conditions characterized by complex interactions between the immune system, epidermal barrier, and environmental factors [1]. Atopic dermatitis, psoriasis, hidradenitis suppurativa, and vitiligo are among the most extensively studied, due to their prevalence, clinical burden, and rapid evolution of targeted therapeutic options. Over the past decade, advances in immunology and molecular biology have reshaped the understanding of these disorders, shifting the field from descriptive morphology to mechanistic and pathway-driven disease models [2,3].

Central to this evolving perspective is the immune microenvironment, which encompasses interactions between keratinocytes, immune cells, cytokines, and structural components (extracellular matrix). Dermatopathology has traditionally focused on identifying diagnostic patterns such as spongiosis in atopic dermatitis, acanthosis and parakeratosis in psoriasis, follicular occlusion in hidradenitis suppurativa, and melanocyte loss in vitiligo. These features are not only diagnostically useful but also reflect disease-specific immune mechanisms that increasingly inform therapeutic targeting across inflammatory dermatoses [1,2,3,4,5].

These four diseases were selected as representative models due to their well-characterized immune pathways and availability of targeted therapies, allowing the integration of histopathological and molecular perspectives.

Although the immune mechanisms underlying these diseases have been extensively reviewed, their direct translation into practical histopathological interpretation remains less consistently emphasized. This review, therefore, aims not only to summarize immune pathways but also to highlight how specific microscopic patterns may serve as tissue-level reflections of cytokine networks, immune cell recruitment, epithelial dysfunction, and therapeutic targets. By integrating morphology, immune mechanisms, and treatment implications, the review seeks to provide a practical framework for dermatopathological orientation in selected inflammatory skin diseases.

## 2. Materials and Methods

This study was designed as a narrative review to integrate current evidence on the cutaneous immune microenvironment in major inflammatory skin diseases. A literature search was conducted using PubMed, Scopus, and Web of Science, focusing on recent publications addressing immune mechanisms, histopathology, and targeted therapies. The search was completed in March 2026.

The literature search included combinations of the following keywords: “cutaneous immune microenvironment”, “dermatopathology”, “atopic dermatitis”, “psoriasis”, “hidradenitis suppurativa”, “vitiligo”, “cytokines”, “immune pathways”, “histopathology”, “targeted therapy”, and “JAK inhibitors”.

Original articles, translational studies, clinical studies, and selected review articles published in English between 2021 and early 2026 were considered. The selected time frame was chosen to emphasize recent advances in immune-mediated mechanisms and targeted therapies while incorporating representative earlier studies when necessary for mechanistic or historical context. Articles were selected based on their relevance to the integration of histopathological findings, immune pathways, and therapeutic implications.

Given the heterogeneity of the available literature, including mechanistic studies, histopathological descriptions, and therapeutic data, a formal systematic review protocol with predefined inclusion and exclusion criteria was not applied. Instead, a narrative approach was used to enable integration of diverse evidence and to support a clinically oriented discussion.

This approach allows flexibility in synthesizing recent advances but may introduce selection bias and limit reproducibility. In addition, no formal meta-analysis was performed; therefore, quantitative estimates of the prevalence of histopathological features were not generated. Where possible, semi-quantitative descriptors were included to enhance practical diagnostic interpretation.

## 3. The Cutaneous Immune Microenvironment

### 3.1. Cellular Components

The cutaneous immune microenvironment consists of a highly coordinated network of cells, including keratinocytes, dendritic cells, macrophages, and T lymphocytes. T lymphocytes play a central role in coordinating disease-specific immune responses, with distinct subtypes such as T helper 1 (Th1), T helper 2 (Th2), and T helper 17 (Th17) driving various inflammatory phenotypes. In addition to circulating immune cells, tissue-resident memory T cells contribute to the persistence and recurrence of inflammation, emphasizing the importance of local immune regulation. Keratinocytes act as immune modulators, producing cytokines and chemokines that influence immune cell recruitment and activation, reinforcing the concept of the skin as an active immune organ rather than merely a passive barrier [1,5].

### 3.2. Cytokine Network

Cytokines represent the central elements of the immune microenvironment, defining disease-specific inflammatory hallmarks. Distinct cytokine axes, including Th2-associated cytokines (IL-4, IL-13), the IL-17, IL-23 axis, and cytotoxic IFN-γ-mediated pathways, form the core of major inflammatory skin diseases discussed in this review. The IL-4/IL-13 axis signals through JAK-dependent pathways with downstream activation of STAT6, which is central to type 2 inflammation, while IL-17 and IL-23 drive keratinocyte activation and chronic inflammation in psoriasis. In parallel, neuroimmune mechanisms have emerged as important modulators of cytokine signaling, linking neuronal pathways to immune activation and contributing to disease symptoms such as pruritus [2,6].

### 3.3. Barrier and Tissue Interaction

The epidermal barrier plays a fundamental role in maintaining immune homeostasis. Disruption of its integrity facilitates the penetration of allergens, microbes, and environmental triggers, leading to immune activation and chronic inflammation. In atopic dermatitis, alterations in epidermal lipids and barrier proteins create a permissive environment for microbial colonization, allergen penetration, and sustained immune activation [3,4].

Beyond barrier dysfunction, extracellular matrix components actively influence immune signaling. Structural alterations in keratinocytes and matrix remodeling contribute to disease progression and chronic inflammation, particularly in conditions such as hidradenitis suppurativa. Their interactions underscore the bidirectional relationship between tissue structure and immune function [2,4].

In this context, dermatopathology is positioned to bridge morphology and molecular biology. Tissue-based analyses provide direct insight into the cellular and cytokine composition of inflammatory lesions, enabling a deeper understanding of disease mechanisms and therapeutic targets. As targeted therapies continue to emerge, integrating histopathological findings with molecular pathways is essential for advancing precision dermatology [2,7].

## 4. Atopic Dermatitis: Th2-Dominated Microenvironment

Atopic dermatitis is an inflammatory skin disease characterized by a complex interplay between epidermal barrier dysfunction and immune dysregulation. The cutaneous immune microenvironment is predominantly driven by type 2 immune responses (mediated by Th2) with additional involvement of Th22 and Th1 pathways in chronic stages. This immunological profile is reflected not only in circulating biomarkers but also in histopathological and molecular features observed within lesional skin [1,3].

### 4.1. Histopathology

The histopathological pattern of atopic dermatitis varies according to lesion stage. Acute lesions are dominated by spongiosis, which is usually the most prominent epidermal alteration and may be accompanied by exocytosis of lymphocytes. In subacute lesions, spongiosis persists but is associated with increasing acanthosis and parakeratosis. Chronic lesions are characterized by irregular epidermal hyperplasia, compact hyperkeratosis, variable parakeratosis, and only mild residual spongiosis [3,4].

Spongiosis represents the most consistent finding in acute lesions and is observed in most biopsies, whereas eosinophils are variably present and depend on disease stage and severity. In the dermis, a superficial perivascular inflammatory infiltrate composed mainly of lymphocytes is typically observed. Eosinophils may be present, although their number is variable and they are not required for diagnosis. Their presence supports a type 2 inflammatory background, particularly in clinically compatible lesions. Additional findings may include papillary dermal edema in acute lesions and dermal fibrosis or lichenification-related changes in chronic disease [4,5].

From a diagnostic perspective, the combination of spongiosis, superficial perivascular lymphocytic inflammation, variable eosinophils, and chronic epidermal remodeling is highly suggestive of atopic dermatitis in the appropriate clinical context. These morphological findings reflect the underlying Th2-skewed cytokine milieu, particularly IL-4 and IL-13 signaling, which contributes to impaired epidermal differentiation, barrier dysfunction, and persistent local immune activation [3,5].

### 4.2. Molecular Pathways and Barrier Dysfunction

Atopic dermatitis is driven by a complex interaction between epidermal barrier defects and immune dysregulation, with the IL-4/IL-13–JAK–STAT6 axis representing a central pathogenic pathway. Type 2 inflammatory cytokines, IL-4 and IL-13, signal through JAK-dependent pathways and lead to activation of STAT6. This results in transcriptional repression of essential epidermal differentiation genes such as filaggrin, loricrin, and involucrin, as well as enzymes involved in ceramide synthesis, thereby directly impairing barrier integrity. Furthermore, this cytokine milieu downregulates antimicrobial peptides, facilitating microbial colonization, particularly by Staphylococcus aureus, which amplifies inflammation through superantigen production and Th2 polarization. In parallel, epithelial-derived alarmins such as TSLP, IL-25, and IL-33 activate dendritic cells and group 2 innate lymphoid cells (ILC2s), promoting Th2 differentiation and perpetuating cytokine release [7,8,9].

**Figure 1:**
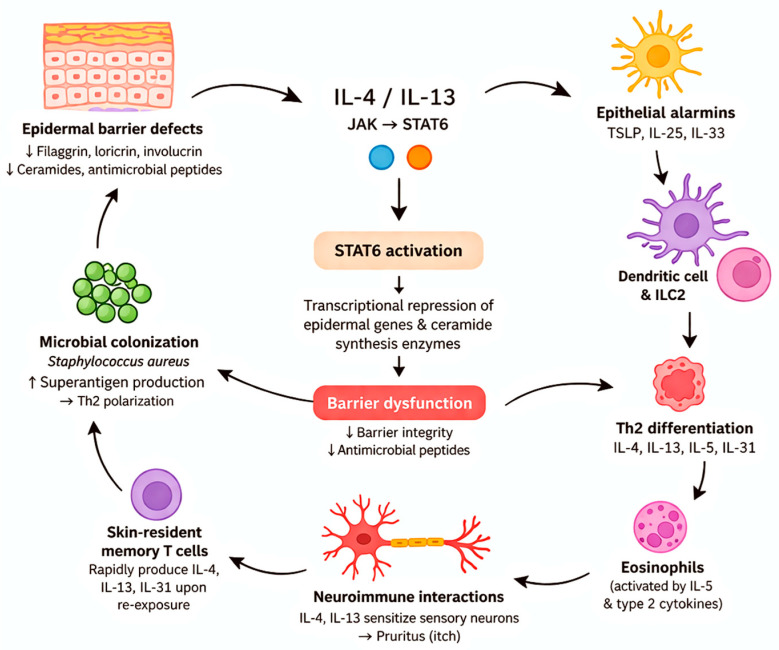
Molecular mechanisms in atopic dermatitis. Schematic representation of the IL-4/IL-13–JAK–STAT6 signaling axis and its effects on keratinocyte differentiation, barrier dysfunction, and immune activation. The role of epithelial-derived cytokines (TSLP, IL-25, IL-33) and microbiome interactions is also illustrated.

Chronic disease is thought to be sustained by skin-resident memory T cells, which rapidly produce type 2 cytokines and contribute to recurrent inflammation. IL-4, IL-13, and IL-31, produced upon antigen re-exposure, maintain inflammation even in clinically normal skin. Additionally, neuroimmune interactions also contribute, as IL-4 and IL-13 directly sensitize sensory neurons and enhance pruritogenic signaling. Eosinophils, activated downstream of IL-5 and type 2 cytokines, further contribute to tissue remodeling and inflammation. Collectively, these mechanisms establish a self-amplifying loop in which barrier dysfunction and type 2 immune responses reciprocally reinforce each other, underlying both disease initiation and chronicity in atopic dermatitis [9,10]. These mechanisms are summarized in Figure 1.

### 4.3. Therapeutic Options

Treatment of atopic dermatitis targets the epidermal barrier dysfunction and type 2 immune dysregulation. Management includes both barrier repair options and targeted immunomodulation, with emollients and ceramide-based moisturizers, which restore lipid composition, reduce transepidermal water loss, and limit allergen and microbial penetration. Topical anti-inflammatory therapies, including corticosteroids and calcineurin inhibitors, remain first-line treatments, while topical JAK inhibitors provide targeted inhibition of cytokine signaling downstream of IL-4 and IL-13. In moderate-to-severe disease, systemic immunosuppressants can be used, such as cyclosporine, methotrexate, azathioprine, and mycophenolate mofetil, alongside oral JAK inhibitors that interfere with intracellular signaling pathways central to disease pathogenesis. Biologic therapies targeting type 2 inflammation, including dupilumab, lebrikizumab, and tralokinumab, have shown significant efficacy in moderate-to-severe atopic dermatitis by inhibiting IL-4 and IL-13 signaling [11,12,13,14,15]. These therapies reduce inflammation and contribute to the restoration of barrier function. Emerging strategies include microbiome modulation, neuroimmune pathway targeting, and biomarker-guided treatment selection, reflecting an evolving precision medicine approach in atopic dermatitis. Recent real-world evidence further supports the effectiveness of JAK inhibitors in moderate-to-severe atopic dermatitis, particularly in patients with inadequate response to biologic therapies [11,16,17,18,19].

## 5. Psoriasis: Immunopathogenesis and Cytokine Pathways

Psoriasis is a chronic, immune-mediated disorder with important environmental risk factors that act as triggers. The disease is characterized by hyperproliferation of the epidermis, with a high epidermal turnover rate and scale formation. The disease is driven by interconnected molecular mechanisms that create a self-sustained loop [20].

### 5.1. Histopathology

Psoriasis shows one of the most characteristic psoriasiform reaction patterns in dermatopathology. Fully developed plaques typically demonstrate regular acanthosis with elongated and relatively uniform rete ridges, thinning of the suprapapillary plates, confluent parakeratosis, and reduction or absence of the granular layer. Dilated and tortuous capillaries are frequently present in the papillary dermis and correspond clinically to the Auspitz phenomenon [20].

Neutrophilic collections represent important diagnostic clues. Munro microabscesses, consisting of neutrophils within the stratum corneum, and spongiform pustules of Kogoj, located within the spinous layer, are highly supportive of psoriasis when present. However, these features may be focal and are not identified in all biopsies, particularly in treated or early lesions [20,21].

Early lesions may show less developed psoriasiform hyperplasia and a more prominent superficial perivascular lymphocytic infiltrate, while chronic plaques show the classic combination of regular epidermal hyperplasia, parakeratosis, hypogranulosis, neutrophilic microabscesses, and vascular remodeling. This constellation helps distinguish psoriasis from chronic eczematous dermatitis, pityriasis rubra pilaris, and other psoriasiform dermatoses. The histological pattern closely mirrors activation of the IL-23/Th17 axis, with IL-17- and IL-22-driven keratinocyte hyperproliferation, neutrophil recruitment, and angiogenesis. The most frequent and important findings are represented by the psoriasiform pattern associated with confluent parakeratosis and chronic dermal inflammation. The other characteristics mentioned are not always seen [20,21].

### 5.2. Immune Pathways

Psoriasis is a complex disease with multiple molecules and cells involved that develops through a tightly linked sequence of innate and adaptive immune events in genetically predisposed skin, with the IL-23/Th17 axis acting as the central pathway. Disease initiation is thought to begin when environmental triggers such as trauma, infection, stress, or drugs disturb the epidermal barrier and activate keratinocyte proliferation. Under these triggers, keratinocytes become activated and release antimicrobial peptides, self-nucleic acids, and alarm mediators that are sensed by innate immune cells, particularly the dendritic cells, which act as antigen-presenting cells (APC) [21,22].

Complexes of self-DNA or self-RNA with antimicrobial peptides such as LL-37 can stimulate plasmacytoid dendritic cells, which produce interferons and help create the first inflammatory response. At the same time, myeloid dendritic cells become activated and secrete cytokines, especially IL-12 and IL-23, along with TNF-α and other inflammatory signals. Although earlier models emphasized a Th1-mediated process, current understanding places IL-23-driven pathogenic Th17 responses at the core of the disease. IL-23 promotes the expansion, stabilization, survival, and pathogenic programming of Th17 cells and related IL-17-producing populations, including Tc17 cells, γδ T cells, innate lymphoid cells, and, in some contexts, additional immune cells such as mast cells may also contribute. These populations produce IL-17A, IL-17F, IL-22, TNF-α, and other mediators that act directly on keratinocytes. IL-17 induces a pro-inflammatory state in keratinocytes, which in turn start producing chemokines, cytokines, antimicrobial peptides, and growth factors that amplify leukocyte recruitment and epidermal remodeling [18,19,20,21,22]. IL-22 will stimulate keratinocyte hyperplasia and abnormal differentiation, contributing to acanthosis, parakeratosis, and loss of the granular layer. TNF-α, IL-17, and IL-22 will magnify transcriptional responses in keratinocytes and endothelial cells. As this inflammatory process persists, the lesions become self-sustaining: IL-23 maintains pathogenic lymphocytes, IL-17 and IL-22 reshape the epidermis, TNF-α amplifies the inflammatory network, and angiogenic factors such as VEGF remodel the microvasculature. This mechanistic framework explains the efficacy of biologic therapies targeting TNF, IL-17, and IL-23, which interrupt key nodes in the pathogenic network. These cytokines occupy key positions in the dominant pathogenic circuit described in recent psoriasis literature [22,23,24].

### 5.3. Cellular Interactions

Cell interactions in psoriasis are a tightly coordinated circuit between keratinocytes, dendritic cells, T cells, and innate immune populations. The process is initiated by stress-activated keratinocytes, which release antimicrobial peptides, cytokines, and nucleic acid complexes that activate plasmacytoid and myeloid dendritic cells, leading to type I interferon production and subsequent IL-23 and TNF-α secretion [19,24]. These dendritic cell–derived signals drive the differentiation, expansion, and stabilization of pathogenic Th17 cells and other IL-17–producing populations, which become the dominant effector cells in psoriatic lesions [21]. Th17-derived cytokines, particularly IL-17A, IL-17F, and IL-22, act directly on keratinocytes, inducing hyperproliferation, impaired terminal differentiation, and a marked increase in the production of chemokines such as CXCL1, antimicrobial peptides, and pro-inflammatory mediators. This keratinocyte activation amplifies the recruitment of neutrophils and additional CCR6^+^ Th17 cells, reinforcing the inflammatory infiltrate [23,25].

**Figure 2:**
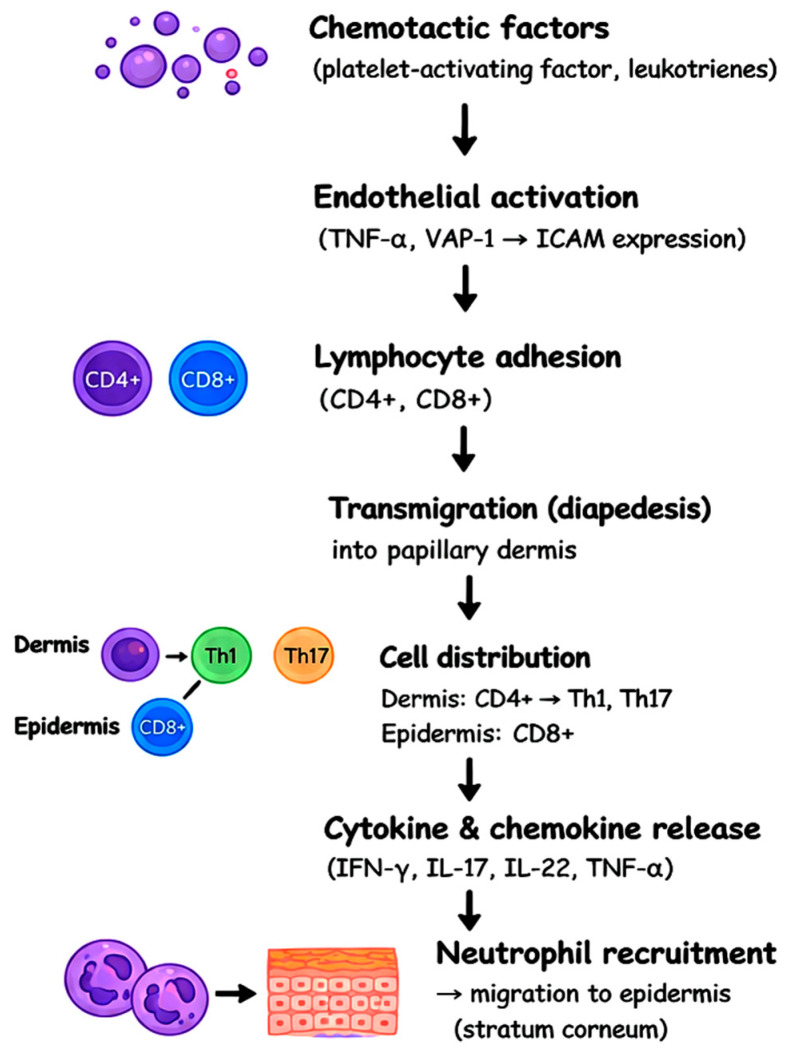
Cellular interactions and cytokine effects in psoriasis. Diagram illustrating the IL-23/Th17 axis and its interaction with keratinocytes, dendritic cells, and neutrophils, highlighting the self-amplifying inflammatory loop.

Neutrophils migrate into the epidermis in response to keratinocyte-derived chemokines, forming characteristic microabscesses (Munro abscesses) and contributing to inflammation through proteases, reactive oxygen species, and extracellular traps, which stimulate innate immune pathways [26]. Factors such as VEGF induce endothelial activation, vascular dilation, and neovascularization in the papillary dermis, facilitating sustained leukocyte migration into the skin [27]. Throughout this process, continuous exchange between keratinocytes and immune cells maintains a loop in which IL-23 sustains pathogenic T cells, IL-17 and IL-22 perpetuate epidermal alterations, and TNF-α amplifies inflammation across multiple cell types. Increasing real-world evidence also supports the durable efficacy and favorable long-term disease control achieved with selective IL-23 inhibitors [27,28]. All these mechanisms are highlighted in Figure 2.

### 5.4. Therapeutic Options

Therapeutic options in psoriasis have evolved with the understanding of its pathogenesis, shifting from nonspecific immunosuppression toward targeted modulation of inflammatory pathways. Topical corticosteroids, vitamin D analogues, and phototherapy remain essential for mild to moderate disease. These methods reduce keratinocyte proliferation and local inflammation, but their effects are largely symptomatic. In severe or refractory cases, systemic agents such as methotrexate, cyclosporine, and acitretin are used to broadly suppress immune activation and keratinocyte turnover, but their long-term use is limited by toxicity and lack of pathway specificity [28].

The identification of the IL-23/Th17 axis as a central driver of psoriasis has led to the development of biologic therapies that selectively interrupt cytokine networks. Tumor necrosis factor (TNF) inhibitors were among the first targeted agents and remain effective due to their broad role in all stages of the inflammatory signaling. Therapies targeting IL-17 (monoclonal antibodies against IL-17A or its receptor) have shown rapid and efficient clinical responses by directly suppressing keratinocyte activation and neutrophil recruitment, which are key downstream effects of Th17 immunity. Furthermore, increasing evidence supports IL-23 as an upstream regulator of pathogenic T-cell populations; thus, selective IL-23 inhibitors have also shown high efficacy with durable responses, likely by disrupting the maintenance of disease-driving Th17 cells [29,30,31,32].

Beyond cytokine blockade, new therapies aim to restore immune and barrier homeostasis through alternative mechanisms. For example, aryl hydrocarbon receptor modulators such as tapinarof influence keratinocyte differentiation, oxidative stress, and inflammatory gene expression, representing a non-immunosuppressive approach that targets both epithelial and immune components of the disease [33,34]. Psoriasis is increasingly recognized as a systemic inflammatory condition associated with comorbidities such as cardiometabolic disease, which has influenced treatment goals toward achieving sustained disease control and potentially modifying long-term inflammatory burden [34,35]. Current research is therefore focused not only on achieving skin clearance but also on improving the durability of response and exploring the possibility of longer-term disease modification through early and targeted intervention [36,37].

Modern psoriasis therapy reflects a transition from generalized immunosuppression to precision targeting of disease-relevant pathways, with ongoing efforts for durable remission.

## 6. Hidradenitis Suppurativa: Epithelial Dysfunction and Neutrophil-Rich Inflammation

Hidradenitis suppurativa is a chronic inflammatory disease characterized by painful nodules, abscesses, scarring, and a complex molecular and cellular landscape [38].

### 6.1. Histopathology

Hidradenitis suppurativa is characterized by a folliculocentric inflammatory pattern with stage-dependent histological changes. Early lesions show infundibular hyperkeratosis, follicular plugging, and follicular dilatation. These changes support the concept that follicular occlusion represents an initiating event in the disease. Progression leads to follicular rupture, with release of keratin, hair shaft fragments, and microbial products into the dermis [38,39].

Ruptured follicles trigger a dense mixed inflammatory infiltrate composed of neutrophils, lymphocytes, plasma cells, macrophages, and foreign-body giant cells. These are frequently seen in early lesions. Neutrophil-rich abscess formation is common in active lesions and correlates with the intense innate immune activation observed in hidradenitis suppurativa. In more advanced lesions, the most characteristic and frequent histological finding is the presence of epithelium-lined sinus tracts or dermal tunnels, usually associated with chronic inflammation, granulation tissue, fibrosis, and scarring [38].

From a diagnostic standpoint, follicular occlusion, follicular rupture, abscess formation, and epithelialized tunnels are key clues. The presence of dermal tunnels is particularly useful in established diseases and distinguishes hidradenitis suppurativa from simple folliculitis or isolated abscesses. These histological features reflect the integration of epithelial dysfunction, neutrophil-rich inflammation, microbial persistence, and stromal remodeling [38].

### 6.2. Molecular Mechanisms

**Figure 3:**
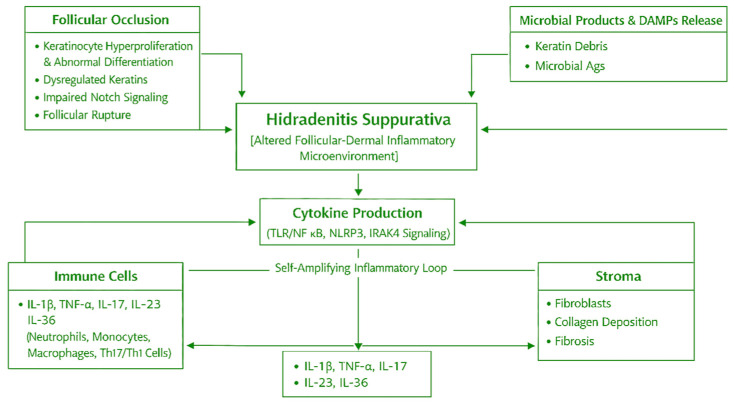
Mechanisms in hidradenitis suppurativa. Representation of follicular occlusion, innate immune activation, cytokine signaling (IL-1β, TNF-α, IL-17), and the role of neutrophils and microbiome dysbiosis.

Hidradenitis suppurativa is characterized by an altered follicular–dermal inflammatory microenvironment in which epithelial, immune, microbial, and stromal components form a self-amplifying loop. Disease initiation occurs through follicular occlusion caused by keratinocyte hyperproliferation and abnormal differentiation, associated with dysregulated keratins and impaired Notch signaling. These changes lead to follicular rupture and release of keratin debris, microbial products, and damage-associated molecular patterns (DAMPs). These signals activate innate immune pathways (TLR/NF-κB, NLRP3 inflammasome, IRAK4 signaling) within keratinocytes and immune cells, resulting in a cytokine-rich milieu. The most important cytokines are IL-1β, TNF-α, IL-17, IL-23, and IL-36, and they control the recruitment of neutrophils, monocytes, and macrophages, and Th17/Th1 lymphocytes [39]. These mechanisms are summarized in Figure 3.

Neutrophils contribute to tissue injury through proteases, reactive oxygen species, and neutrophilic extracellular traps (NETs). Macrophages and T cells sustain chronic inflammation by secreting cytokines and creating feedback loops. Microbiome dysbiosis and biofilm formation, particularly within sinus tracts, are thought to contribute to persistent antigenic stimulation and further reinforce innate immune activation. Keratinocytes within lesional epidermis and tunnel linings acquire an aberrant, hyperproliferative and proinflammatory phenotype, actively producing cytokines and perpetuating epithelial–immune crosstalk. In parallel, the stromal compartment undergoes extensive remodeling, characterized by fibroblast activation, matrix metalloproteinase activity, altered extracellular matrix signaling, and progressive fibrosis. The resulting epithelium-lined tunnels act as immunologically active niches, maintaining chronic inflammation and impairing resolution [40,41].

Thus, hidradenitis represents a reprogrammed cutaneous microenvironment in which epithelial dysfunction, immune dysregulation, microbial persistence, and stromal remodeling are tightly integrated [40,41,42].

### 6.3. Treatment Options

The management of hidradenitis suppurativa increasingly follows a pathogenesis-driven approach that targets the inflammatory, microbial, and structural components of the disease microenvironment. In mild disease, treatment relies on topical and systemic anti-inflammatory and antimicrobial therapies, including topical clindamycin and oral tetracyclines or combination regimens such as clindamycin–rifampicin, which help reduce bacterial load and secondary immune activation. In moderate-to-severe disease, treatment includes targeted biologic agents, notably TNF-α inhibition with adalimumab, and IL-17A inhibition with secukinumab, while additional therapeutic strategies directed against the IL-17/IL-23 pathway, IL-1 signaling, and IL-36 and innate immune signaling pathways remain under investigation [42,43]. Increasing attention is also given to modulating innate immune signaling pathways, such as IRAK4 inhibition, and to strategies targeting neutrophil activity, given their contribution to tissue damage and chronic inflammation. Adjunctive approaches include hormonal therapies, retinoids, and, in selected cases, immunosuppressants, while microbiome-targeted interventions aim to disrupt biofilms and reduce persistent antigenic stimulation. In advanced diseases with tunnel formation and fibrosis, surgical management remains essential, often combined with medical therapy to control inflammation and prevent recurrence [40,44,45,46,47].

## 7. Vitiligo: Cytotoxic T Cell-Mediated Destruction

Vitiligo is an immune-mediated disease characterized by the destruction of melanocytes. Although several aspects remain unclear, immune mechanisms are central to vitiligo pathogenesis [46].

### 7.1. Histopathology

The histopathological findings in vitiligo depend strongly on disease activity and lesion stage. Established lesions are characterized by marked reduction or complete absence of melanocytes from the basal layer. This finding may be subtle on routine hematoxylin and eosin staining and is best confirmed using melanocytic markers such as Melan-A, SOX10, or HMB-45. Complete melanocyte loss is consistently observed in established lesions, while inflammatory changes are more variable and typically confined to active margins [48].

Active margins may show mild interface dermatitis, focal basal vacuolar change, pigment incontinence, superficial perivascular or lichenoid lymphocytic inflammation, and scattered melanophages in the papillary dermis. The inflammatory infiltrate is usually composed predominantly of T lymphocytes, with CD8-positive cytotoxic T cells playing a central pathogenic role. In stable or long-standing lesions, inflammation may be minimal or absent, and the epidermis can appear relatively preserved apart from melanocyte loss [48,49].

The most useful diagnostic clue is the reduction or absence of melanocytes in a clinically compatible depigmented lesion. Evaluation of the active border is particularly informative because it may reveal the immune-mediated interface changes that are absent from the center of stable lesions. These findings reflect cytotoxic T-cell-mediated melanocyte destruction and IFN-γ/JAK-STAT-driven recruitment of autoreactive lymphocytes [48,49].

### 7.2. Molecular and Pathophysiological Mechanisms

**Figure 4:**
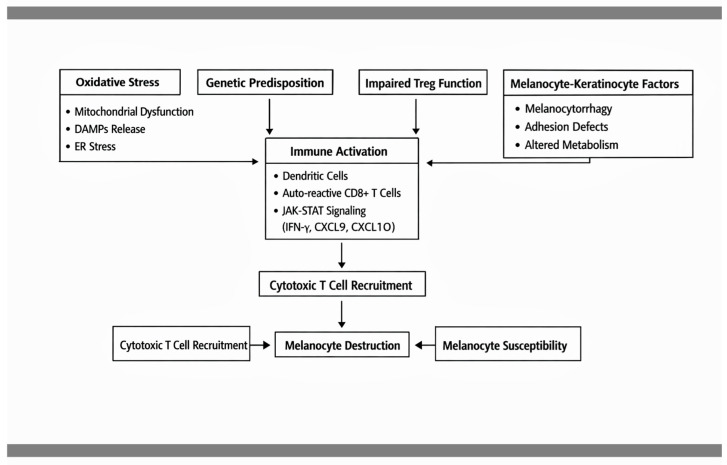
Molecular mechanisms in vitiligo. Overview of IFN-γ–mediated JAK-STAT signaling, CD8+ T-cell–mediated melanocyte destruction, and chemokine-driven immune cell recruitment.

Vitiligo arises from the interplay between intrinsic melanocyte vulnerability and dysregulated immune responses. Oxidative stress is considered a key initiating factor, leading to melanocyte damage, mitochondrial dysfunction, and the release of danger-associated molecular patterns (DAMPs), which activate innate immunity. This triggers dendritic cell activation and subsequent priming of autoreactive CD8+ T cells. A central pathogenic axis involves interferon-γ–mediated signaling through the JAK-STAT pathway, inducing chemokines such as CXCL9 and CXCL10 that recruit additional cytotoxic T cells to the skin. Genetic predisposition contributes through polymorphisms in immune-regulatory and melanocyte-related genes. Additionally, impaired regulatory T-cell function fails to suppress autoreactivity, while keratinocyte–melanocyte crosstalk amplifies local inflammation. Non-immune mechanisms also play a role, including defective melanocyte adhesion (melanocytorrhagy), endoplasmic reticulum stress, and altered cellular metabolism, all of which increase melanocyte susceptibility to immune-mediated destruction [50,51,52]. These mechanisms are briefly represented in Figure 4.

### 7.3. Treatment Options

Therapeutic strategies in vitiligo increasingly reflect its underlying molecular mechanisms. Conventional treatments such as topical corticosteroids and calcineurin inhibitors remain first-line for reducing local inflammation and halting disease progression. Narrowband UVB phototherapy is a cornerstone modality that promotes melanocyte proliferation, migration from hair follicles, and immunomodulation by suppressing pathogenic T-cell responses. Advances in understanding the interferon-γ/JAK-STAT axis have led to clinical use of JAK inhibitors in vitiligo, most notably with topical ruxolitinib for nonsegmental vitiligo, while systemic JAK inhibitors remain under investigation or off-label in many settings. Combination therapies, particularly JAK inhibitors with phototherapy, appear to enhance efficacy and durability of response. Additional approaches include antioxidant therapies that reduce oxidative stress, as well as emerging strategies targeting resident memory T cells to prevent relapse. Overall, modern vitiligo management is shifting toward targeted, mechanism-based interventions designed not only to induce repigmentation but also to maintain long-term disease control [53,54].

## 8. From Morphology to Therapy

The transition from descriptive histopathology to mechanism-driven treatments represents a major shift in dermatopathology. The morphological patterns of the diseases reflect distinct immunologic markers that can be therapeutically targeted. To facilitate practical diagnostic orientation, the main histopathological patterns, key diagnostic clues, and stage-dependent features of the selected diseases are summarized in Table 1. This summary may also assist in the differential diagnosis between these entities and their main histopathological mimickers, particularly in limited or partially treated biopsies.

In atopic dermatitis, the most characteristic features are spongiosis, epidermal barrier disruption, and a superficial perivascular lymphocytic infiltrate. These features correspond to a type 2 immune response, where cytokines IL-4 and IL-13 activate downstream signaling pathways. These morphological and molecular features directly underpin the development of targeted drugs such as dupilumab, tralokinumab, and lebrikizumab. These therapies target key type 2 inflammatory pathways: dupilumab inhibits signaling through IL-4Rα, thereby affecting both IL-4 and IL-13 pathways, whereas tralokinumab and lebrikizumab selectively target IL-13 [1,5,6].

The emergence of JAK inhibitors further expands therapeutic options by interfering with intracellular cytokine signaling pathways [10].

In psoriasis, histopathological findings include acanthosis, parakeratosis, neutrophilic microabscesses, and prominent dermal vascular proliferation. These reflect activation of the IL-23/Th17 axis. Crosstalk between keratinocytes and immune cells sustains a pro-inflammatory loop involving IL-17, IL-22, and TNF-α. The presence of angiogenesis and systemic inflammatory features further highlights psoriasis as a multisystemic disease. These insights have led to highly effective targeted therapies, including IL-17 and IL-23 inhibitors, as well as novel agents, such as bimekizumab and tapinarof. Importantly, these treatments not only improve clinical lesions but also influence the underlying inflammatory pathways, suggesting a potential role in disease modification [25,28,30].

In hidradenitis suppurativa, the morphological spectrum is characterized by follicular occlusion, sinus tract formation, and a dense inflammatory infiltrate, rich in neutrophils and macrophages. Dysregulated keratinization and extracellular matrix remodeling contribute to the formation of chronic tunnels, while microbial dysbiosis and innate immune activation further amplify inflammation. Cytokine profiling reveals involvement of TNF-α, IL-1, and IL-17 pathways, providing a rationale for targeted therapies. Available and emerging treatments, including biologic agents and therapies targeting neutrophil-related pathways, reflect an increasing ability to align treatment strategies with underlying pathogenetic mechanisms [37,39,42].

In vitiligo, histopathological features such as loss of melanocytes and interface dermatitis correspond to autoimmune-mediated melanocyte destruction, driven by cytotoxic T cells and interferon signaling. The interplay between the innate and adaptive immune responses, as well as oxidative stress, contributes to disease progression. These pathophysiological insights have made possible the development of targeted therapies such as JAK inhibitors, which interfere with interferon signaling and promote repigmentation [49,50,51].

Overall, morphology and molecular immunology enable a refined classification of inflammatory dermatoses, moving beyond traditional clinicopathologic correlations toward a precision medicine framework. In this context, dermatopathology plays a key role in diagnosis and in identifying therapeutic targets, while also offering potential future biomarkers of treatment selection and response. 

## 9. Future Directions

Future research in inflammatory skin diseases will likely focus on the role of the cutaneous immune microenvironment through an integration of histopathology, molecular profiling, and clinical data. One of the most promising directions is the identification and validation of biomarkers that may help predict disease activity, therapeutic response, and longer-term outcomes. Immunohistochemistry, multiplex staining, and transcriptomic analysis on formalin-fixed-paraffin-embedded samples offer a widely accessible platform for investigations. Advances in spatial biology techniques are expected to further describe the cellular and cytokine interactions within the skin, allowing a better understanding of immune niches and pathophysiological circuits. These technologies can bridge the gap between morphology and molecular function, especially in diseases with high heterogeneity. Along with these, artificial intelligence and digital pathology have the potential to transform dermatopathological practice by allowing quantitative assessment of inflammatory patterns and automated recognition of disease-specific features. From a treatment perspective, the future lies in personalized strategies that target not only the cytokine pathways but also memory cells, neuroimmune interactions, and microbiome-related mechanisms. Early intervention and disease-modifying approaches may lead to sustained remission.

Despite significant advances in targeted therapies, clinical response remains heterogeneous across patients, reflecting underlying disease complexity and inter-individual variability in immune pathways. A major unmet need remains the identification of reliable biomarkers capable of predicting disease course and therapeutic response. Current evidence suggests that biomarker validation is still limited, underscoring the necessity for integrative approaches that combine histopathological, molecular, and clinical data to enable true precision medicine in inflammatory skin diseases. 

However, despite their significant research potential, widespread implementation in routine dermatopathology practice remains limited by cost, technical complexity, challenges in standardization, and limited prospective validation.

## 10. Conclusions

The cutaneous immune microenvironment provides a unifying framework that integrates histopathological findings with molecular and therapeutic options for inflammatory skin diseases. Atopic dermatitis, psoriasis, hidradenitis suppurativa, and vitiligo, although clinically distinct, share a common ground in which distinct immune pathways interact with the structural elements of the skin to create specific patterns of inflammation. Dermatopathology lies at the intersection of tissue changes and molecular pathology. Classic histological features are now understood as reflections of underlying pathophysiological mechanisms and cytokine networks. The development of targeted therapies, including biologic and molecular inhibitors, further highlights the importance of correlating histopathological patterns with specific immune pathways. Future integration of histopathologic, molecular, and biomarker profiles may improve disease stratification and potentially support therapeutic decision-making, although many proposed biomarkers still require prospective validation before routine clinical implementation.

## Figures and Tables

**Table 1:** Main histopathological diagnostic clues in selected inflammatory skin diseases.

Disease	Main Histopathological Pattern	Key Diagnostic Clues	Stage-Dependent Findings	Pathogenic Correlation
Atopic dermatitis	Spongiotic dermatitis	Spongiosis, superficial perivascular lymphocytic infiltrate, variable eosinophils	Acute: marked spongiosis; Chronic: acanthosis, hyperkeratosis	Th2 inflammation, IL-4/IL-13 signaling, barrier dysfunction
Psoriasis	Psoriasiform dermatitis	Regular acanthosis, parakeratosis, hypogranulosis, Munro microabscesses	Early lesions may lack classic features; chronic plaques show full pattern	IL-23/Th17 axis, IL-17/IL-22-driven keratinocyte activation
Hidradenitis suppurativa	Folliculocentric suppurative inflammation	Follicular plugging, rupture, abscesses, epithelialized sinus tracts	Early: follicular occlusion; Advanced: tunnels, fibrosis, scarring	Epithelial dysfunction, neutrophil-rich inflammation, TNF/IL-17 pathways
Vitiligo	Melanocytopenic/interface pattern	Loss of melanocytes, CD8+ lymphocytes, pigment incontinence	Active margins show inflammation; stable lesions show melanocyte loss only	Cytotoxic T-cell response, IFN-γ/JAK-STAT signaling

## Data Availability

No new data were created or analyzed in this study. Data sharing is not applicable to this article.
